# Sterile Fluid Transfer for Cell Therapy Manufacturing—The Value of Multiple-Use Aseptic Connector

**DOI:** 10.3389/fbioe.2021.806677

**Published:** 2022-03-04

**Authors:** Ying Ying Wu, Jia Sheng Zach Lee, Sixun Chen, Dan Liu

**Affiliations:** Bioprocessing Technology Institute, A*STAR Research Entities, Singapore, Singapore

**Keywords:** cell manufacturing, sterile fluid transfer, aseptic fluid handling, sterile connector, closed system

## Abstract

Sterile fluid transfer between vessels is a key step in cell therapy manufacturing and requires specialised devices to maintain sterility of both vessels before, during and after the transfer. This review introduces two main types of devices for sterile fluid transfer in cell therapy manufacturing, namely single-use sterile connectors and tube welders. While these are excellent devices for infrequent moderate to large volume transfers, a new multiple-use aseptic connector may fill the gap for frequent small volume fluid transfers that are particularly important for autologous cell therapy manufacturing.

## Introduction

Cell therapy is an advanced therapeutic that utilizes live cells in the treatment of ailments (Aijaz et al., 2018). The manufacturing process of cell therapy products is a permutation of several steps including but not limited to acquisition of live cells as starting material, expansion, modification, volume reduction, purification, formulation, fill and finish, storage, and shipping. These steps are considerably more challenging than those in the manufacturing of traditional biopharmaceutics due to the greater sensitivity of live cells, absence of terminal product sterilization, increased complexity in characterization of cells as final products, and greater variability in starting materials (U.S. Food and Drug Administration, 2004; Lipsitz et al., 2016; Clarke and Taylor, 2019).

Cell therapy can be categorized into allogeneic or autologous cell therapy with each following a different manufacturing model. In allogeneic cell therapy, starting material cells are obtained from a donor who is not the patient. The manufacturing process is geared towards scale-up models similar to those used in biopharmaceutics, and is anticipated to generate production batch sizes of 200–2000 L (Pigeau et al., 2018). In this model, cell culture volume is large and manufacturing tools from biopharmaceutics can be adopted. In contrast, for autologous cell therapy, starting material is obtained from the patient and the final cell therapy product is administered back to the same patient. The manufacturing of this patient-specific product utilizes a scale-out model with small cell culture volumes of less than a few litres per product (Bartel et al., 2013). The choice of using allogeneic or autologous cell therapy depends on several considerations such as higher risk of graft-vs-host disease for allogeneic therapies (Zeiser and Blazar, 2018) and variability in starting material for autologous therapies (Bioprocessintl, 2018 ). Currently, eight out of the nine FDA-approved cell therapies are autologous (FDA, 2021).

With autologous cell therapy manufacturing, achieving the required number of cells for a therapeutic dose may require longer culture period due to low starting volume and potentially low quality of starting material (Lamontagne and Fesnak, 2020). With a longer culture period, sterile fluid transfer steps take up a significant proportion of the culture protocol. These fluid transfers include addition of fresh media or cytokines, removal of waste materials, and extraction of samples for at-line or off-line analyses. All fluid transfer steps must be carefully and aseptically conducted to keep the manufacturing processes and products sterile. Any contamination could result in termination of the manufacturing process and failed products leading to unsuccessful treatment for the patient (Giancola et al., 2012). The current developments in cell therapy manufacturing technologies are shifting towards manufacturing within closed systems via sterile fluid transfers (Parsad, 2020; Samuel, 2021).

## Current Solutions for Sterile Fluid Transfers in Cell Therapy Manufacturing

With closed cell therapy manufacturing, small volume sterile fluid transfers across different culture vessels commonly utilize single-use sterile connectors and tube welders. These devices are based on different working mechanisms and different levels of environmental control are required. Nevertheless, both retain the sterility in the closed vessels before, during and after the transfer. These sterile fluid transfer devices are typically designed for tubing with 1/8” inner diameter.

Although swabbable needleless valves may be used in the context of clean environment, they are typically used only for sample extraction and the complementary pieces to the valves are not aseptic. Thus, they are not within the scope of this paper.

### Tube Welder

**FIGURE 1:**
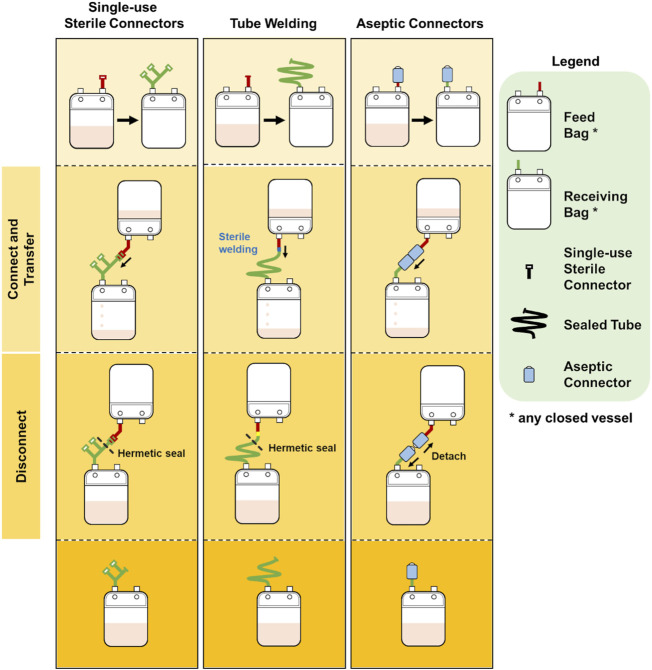
Illustration of the connection and disconnections steps for single-use sterile connector, tube welding and multiple-use aseptic connector.

Briefly, a tube welder connects two tubing by cutting them and fusing the cut ends to allow for fluid flow between the vessels connected to the tubing. After sterile fluid transfer, the two vessels are disconnected by sealing the tubing upstream of the weld and cutting across the weld with scissors. Figure 1 illustrates the use of tube welder to create a sterile connection.

The two tubing are cut with a heat-sterilized metal blade. The metal blade is sterilised by heating to as high as 400°C, and is used to cut both tubing simultaneously. The cut ends are then shifted until they are aligned. Next, the blade is removed and the cut ends are pushed towards each other and allowed to fuse, forming a leak-proof weld. The blade is disposed of after each weld to prevent cross-contamination across welds.

Tube welding has been used for blood processing in blood banks for more than a decade. There are blood bag manifolds pre-configured specifically for common blood processing protocols to minimise the need for tube welding. Used bags are disconnected by tube sealing. If the need to add bags to the manifold arises, tube welding is used to make the connection (Hardwick, 2008). However, there are limitations to applying this method to cell therapy manufacturing, especially since the number of transfers involved in blood processing is far fewer than that in cell therapy manufacturing.

Firstly, although tube welding is performed directly on the tubing and no additional accessory is required, there are some considerations when setting up a culture to use this method of sterile fluid transfer. With every weld, the lengths of tubing are reduced; therefore, planning is required to ensure that there is sufficient tubing length for the total number of welds required in the culture process. Furthermore, as tube welding relies on melting and fusing tubing, it can only weld tubing made of thermoplastic materials. Tubing to be welded must also be the same size and material to ensure leak-free welds.

In addition to these considerations, tube welding is associated with high capital cost and overhead cost as tube welders validated for cell manufacturing are expensive and require regular calibration and maintenance. They are usually deployed in a cleanroom environment to minimise exposure to particulates and contaminants. To minimise the risk of human error, operators are required to attend specific training session before they are allowed to operate a tube welder.

### Single-Use Sterile Connectors

Single-use sterile connectors are accessories attached to the ends of tubing that engages with another complementary single-use sterile connector to create a sterile fluid pathway. Once a connection is established and fluid transfer is completed, the exhausted single-use sterile connector is removed by sealing upstream of the connector with a tube sealer. Figure 1 for an illustration of the use of a single-use sterile connector.

In general, each connector has an aseptic barrier that keeps the contents in the vessel it is attached to closed to the external environment. This aseptic barrier is broken upon engagement with its complimentary connector to allow for fluid transfer. There are several types of aseptic barriers in the design of these connectors. For example, AseptiQuik sterile connectors (Colder Products Company, MN, United States) and Kleenpak sterile connectors (Pall Corporation, NY, United States) make use of membranes to create the aseptic barrier. Upon connection, the sterile membranes are irreversibly removed to establish a sterile fluid pathway between the connectors. The Lynx S2S sterile connector (Merck KGaA, Darmstadt, Germany) operates on a similar mechanism, but with a rigid aseptic barrier instead. These connectors can be adapted for use in various processes such as in one-off connection of sterile vessels or integrated into manifold assemblies for more complex fluid routing. Fail-safe features designed into the connectors and a shift towards genderless connectors minimise the risk of human error and need for operator training.

A key advantage of using single-use sterile connector is its ability to connect tubing of different material and sizes. Generally, companies that develop connectors offer a suite of connectors that can accommodate a wide range of tubing sizes while being compatible with each other. This allows sterile connection to be made across connectors attached to tubing of different sizes.

With single-use sterile connectors, processes with multiple sterile transfers would require a bulky manifold assembly with extensive branches to hold a pre-set number of connectors that satisfies the number of sterile transfers needed. For autologous cell therapy manufacturing, the number of sterile transfers can be high and the manifold assembly might become difficult to manage with numerous connectors. This limits flexibility in modifying the culture protocol during production and requires meticulous planning to ensure that there are sufficient connectors to execute the protocol and cater for contingencies. Extensive manifolds are also costly to manufacture and may result in wastage if fewer connections were utilised.

### Tube Sealer and Tubing Clip

For both tube welders and single-use sterile connectors, after sterile transfer of fluid, the vessels are disconnected using a tube sealer or tubing clip. A tube sealer has two heated metal components that are brought together to compress and permanently fuse the tubing across its lumen, forming hermetic seal. The tubing is then cut across the sealed portion to disconnect the vessels without compromising their sterility. As tube sealing involves melting and fusing tubing, it can only seal thermoplastic tubing. Tubing made of other material may use tubing clips to create the hermetic seal.

### Other Sterile Connectors (Large Volume Sterile Transfer)

There are other sterile connectors currently used in biotherapeutics such as the Lynx CDR sterile connector (Merck KGaA, Darmstadt, Germany). The Lynx CDR enables up to six times of sterile connections, disconnections and reconnections. This ability to reconnect is convenient as it reduces the need to design manifold assembly or welding sites to cater for all possible scenarios during manufacturing. However, it is designed for large volume transfers via tubing with inner diameter of at least 3/8”. Its intended application is biotechnology where scale-up manufacturing involves infrequent sterile fluid transfers in large volumes. For it to be re-designed for autologous cell therapy manufacturing, it needs to be significantly miniaturised and this can be challenging.

## New Sterile Fluid Transfer Device: Multiple-Use Aseptic Connector

We identified a gap in the market for a novel small volume sterile fluid transfer device for autologous cell therapy manufacturing with unique features and termed it ‘multiple-use aseptic connector’. The required features of a multiple-use aseptic connector and comparison to single-use sterile connector and tube welding are tabulated in Table 1. Similar to the Lynx CDR, it should enable multiple sterile connections, disconnections and re-connections without compromising the sterility of the vessels. In addition, to cater for autologous cell therapy manufacturing, this multiple-use aseptic connector has to be designed for sterile transfers of small volumes ranging from tens to several hundreds of millilitres.

**TABLE 1:** Comparison of features across different methods of sterile fluid transfer.

Features	Multiple-use aseptic connector	Single-use sterile connector	Tube welding
Sterile connection	Sterile connection validated by manufacturer	Sterile connection validated by manufacturer	Sterile connection only if specified tubing materials and sizes are used as recommended by manufacturer
Number of connection	Up to a validated number of connections per connector	One-time connection per connector	Number of connections limited by tubing length available for welding
System flexibility	Sterile re-connection allows ad-hoc increase in number of sterile connections	Manifolds are pre-assembled with fixed number of connectors for the same fixed number of sterile connections	Number of connections limited by tubing length available for welding. Welding can only be done between tubing of the same material and size
Tubing selection	Suitable for connecting tubing of any material and a range of sizes	Suitable for connecting tubing of any material and a range of sizes	Limited to tubing of thermoplastic material(s) and size(s) validated for the equipment
Operator training	Minimal training required and low risk of human error	Minimal training required and low risk of human error	Equipment training required with potential for human error
Environment	Connectors designed to be connected under room condition	Connectors designed to be connected under room condition	Equipment recommended to be used in cleanroom environment
Capital cost	No capital and maintenance costs	No capital and maintenance costs	High equipment cost with recurring maintenance cost
Consumables cost	One connector for multiple connections	Every connection requires a new connector	New blade for every weld
Disconnection mechanism	Disconnect flow by disconnecting the multiple-use aseptic connector	Tube sealer or tubing clip used upstream to the connection or welding site	Tube sealer or tubing clip used upstream to the connection or welding site

Multiple-use aseptic connectors would offer the same and more advantages as single-use sterile connectors for cell therapy manufacturing. Firstly, they enable sterile transfer of fluid across tubing of any material and size. Secondly, they are designed to be easy to use and to minimise human error via fail-safe features such as asymmetrical mating features to ensure that there is only one way to connect them. Many single-use sterile connectors are genderless connectors that eliminate handling errors and simplify manifold designs.

In addition, multiple-use aseptic connectors would facilitate more than one sterile transfer by one connector. One multiple-use aseptic connector functionally replaces several single-use sterile connectors thereby eliminating the need for complex and bulky manifolds. This is especially relevant to autologous cell therapy manufacturing involving long culture periods with multiple small volume sterile fluid transfers. The simpler and smaller manifolds that can be achieved with multiple-use aseptic connectors would occupy less space and enable more cultures to be run in the same facility. Figure 1 illustrates the use of a multiple-use aseptic connector.

Consequently, multiple-use aseptic connector would increase system flexibility by enabling users to perform additional sterile transfers for corrective actions such as an additional media top-up without modifying the existing setup. With autologous cell therapy manufacturing, the quality of the starting material is highly varied and protocols may change in response to unexpected culture conditions such as growth rate. Having the option to make additional unforeseen sterile transfers eliminates the need to over-engineer the initial manifold with more single-use sterile connectors or longer tubing lengths. Over-engineered manifolds require more components, and are therefore more costly to assemble, sterilise and transport. They also have a bigger footprint and take up expensive square footage for both deployment and storage in the manufacturing facility. During process development, the flexibility in making additional sterile transfers may shorten process development time by enabling changes in protocol even within the same culture run.

## Value of Multiple-Use Aseptic Connectors

To illustrate the value of a multiple-use aseptic connector, the three different methods of sterile fluid transfer were applied to established manufacturing protocols, and compared and discussed in terms of manufacturing efficiency. Two separate published current Good Manufacturing Practice (cGMP) manufacturing protocols for cell therapy clinical trials involving routine fluid transfers (protocol 1: NCT01807468 and protocol 2: NCT00990717) were used in this value analysis. These protocols were selected due to their utilization of closed culture systems adaptable to sterile transfer technology such as culture bags.

**FIGURE 2:**
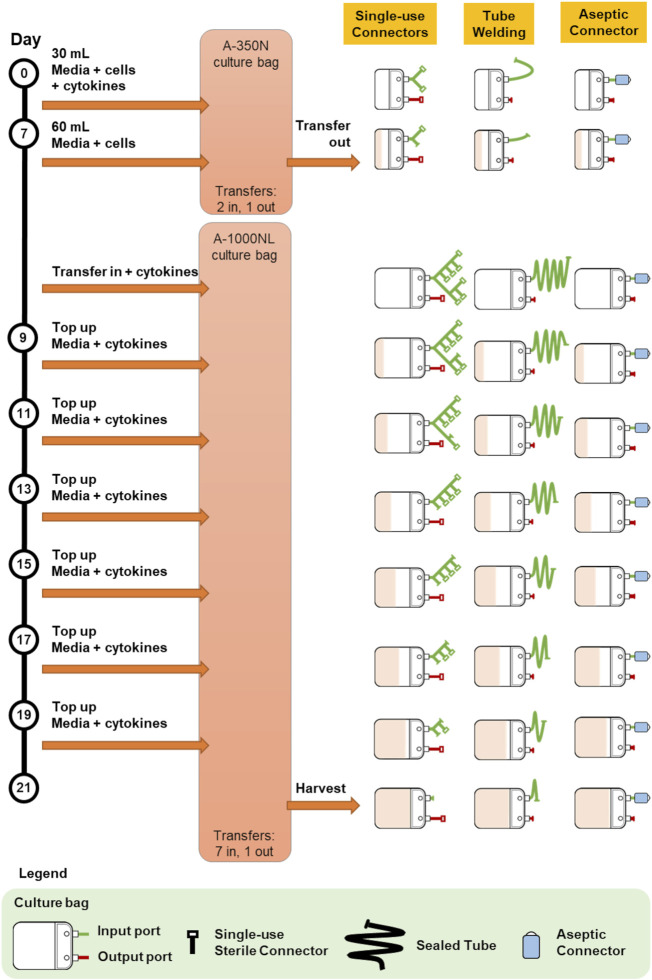
Comparison of bag configurations at each step of protocol 1 NCT01807468 when different sterile fluid transfer methods were used.

Protocol 1 describes the manufacturing of primary NK cells in two different culture bags, A-350N (NIPRO, Osaka, Japan) and A-1000NL (NIPRO, Osaka, Japan). Cells were seeded into A-350N bag and cultured for 7 days, after which media was topped up into the culture and the entire cell culture was transferred to A-1000NL bag. The cells were maintained and fed fresh media every 2 days until the culture reaches 21 days. The use of the three different methods of sterile fluid transfer in this protocol is shown in Figure 2.

**FIGURE 3:**
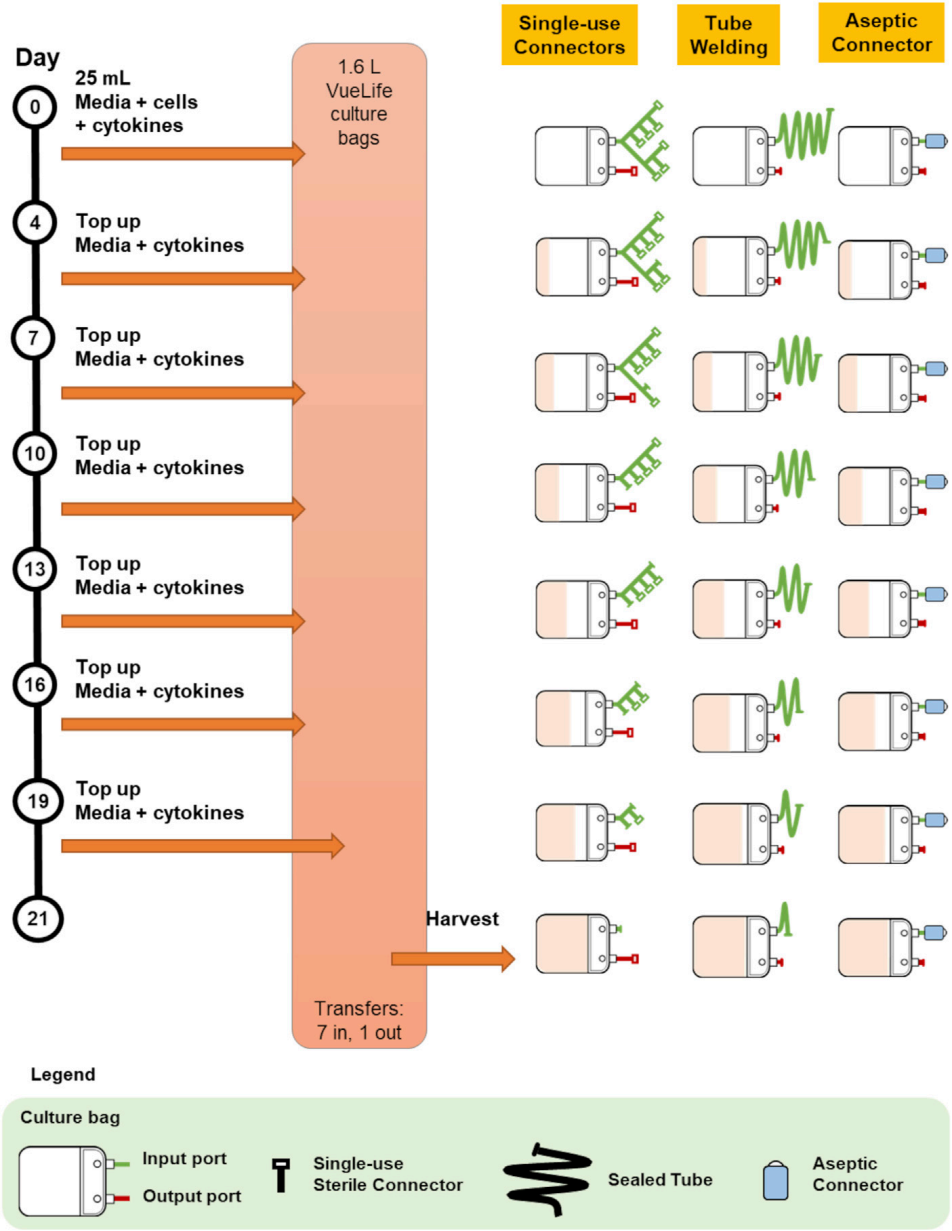
Comparison of bag configurations at each step of protocol 2 NCT00990717 when different sterile fluid transfer methods were used.

Protocol 2 describes the manufacturing of NK-92 cells in 1.6L VueLife culture bag (Saint-Gobain Cell Therapy, Gaithersburg, MD, United States). Cells were seeded into the bag and maintained with routine media top-up step every 3 days for 21 days. The use of the three different methods of sterile fluid transfer in this protocol is shown in Figure 3.

Overall, both protocols involved a maximum of seven fluid input steps per culture bag for the expansion process. The use of single-use sterile connectors required a manifold with sufficient number of connectors to accommodate several media top-up steps. While the illustrations represented each connector as a relatively small device, each connector is about 5 cm wide and with approximately 10 cm of tubing between connectors. Therefore, with every additional connector on the manifold, the bulkiness of the manifold will rapidly increase. For protocols 1 and 2 which require seven single-use connectors for at least one culture bag, approximately 70 cm of tubing or 350 cm^2^ of manifold space (depending on the arrangement and organisation) is needed. This is illustrated by the extended network of single-use connectors in Figure 2 and Figure 3. Moreover, back-up connectors may be required to cater for contingencies, thus further increasing the size of the manifold.

Similar consideration has to be given to the lengths of tubing when tube welder is used as welding have to be done some distance away from previous weld to avoid compromising weld integrity. Both methods result in a bulky attachment to the culture bag. Whereas, in the case of multiple-use aseptic connector, the manifold remains the same regardless of the number of media top-up steps, and this could lead to potential space savings in manufacturing facilities.

The difference in the number of fluid transfers in A-350N (two fluid in steps) and A-1000NL (seven fluid input steps) in Protocol 1 also brought to attention the costs associated with customising culture bags for each protocol. Culture bags with single-use sterile connectors requires customized manifold specific to the protocol while culture bags compatible with tube welder requires bags with sufficiently long tubing lengths. In contrast, the simplicity and flexibility of culture bags with multiple-use aseptic connector is evident as the setup remains the same regardless of the number of transfers required. The simplicity of culture bags with multiple-use aseptic connector would also minimise the risk of human error, as every handling step would be identical.

## Challenge in Materialising the Multiple-Use Aseptic Connector

While developing systems for autologous cell therapy manufacturing, we experienced the challenges of working with single-use sterile connectors and realised the potential benefits of a multiple-use aseptic connector. Thus, we developed a multiple-use aseptic connector (patent pending) and created prototypes for testing and demonstration. Briefly, the prototype was designed as a genderless connector with a diameter of 70 mm and length of 95 mm. It was intended to be used with tubing of 1/8” inner diameter to support small volume sterile fluid transfers. This connector was presented in a poster (Wu and Lee, 2021) and it gathered much interest from end users who are developing new cell therapies.

Despite interest from end users who immediately related this connector to reduced processing time and effort, developing this connector has been a slow process. The first difficulty was in designing mechanisms that enabled re-establishing the sterile barrier after a sterile connection was made. The mechanisms also had to fit within a reasonably small connector size, which made the design complex with several small components. Therefore, the manufacturing of this connector will be complicated and costly in order to achieve the intricate three-dimensional features and tight tolerances. Currently, we are seeking manufacturing partners to productise the connector.

## Conclusion

Sterile transfer of fluids between vessels is a common step in cell therapy manufacturing, particularly in autologous cell therapy manufacturing. Although single-use sterile connectors and tube welders are technically able to perform sterile fluid transfer, they add to wastage of material, bulkiness of cell culture systems, and training and capital costs. The inconvenience and additional costs are more evident in protocols with multiple sterile fluid transfer steps. A new multiple-use aseptic connector as described here would fulfil the unmet need for a sterile connector that can connect, disconnect and re-connect multiple times to transfer small volumes of fluid. Such a technology is especially beneficial for autologous cell therapy manufacturing by reducing the footprint, complexity and cost of culture systems.
